# Facebook, Quality of Life, and Mental Health Outcomes in Post-Disaster Urban Environments: The L’Aquila Earthquake Experience

**DOI:** 10.3389/fpubh.2014.00286

**Published:** 2014-12-22

**Authors:** Francesco Masedu, Monica Mazza, Chiara Di Giovanni, Anna Calvarese, Sergio Tiberti, Vittorio Sconci, Marco Valenti

**Affiliations:** ^1^Section of Environmental Medicine and Clinical Epidemiology, Department of Applied Clinical Sciences and Biotechnology, University of L’Aquila, L’Aquila, Italy; ^2^Section of Neuropsychology, Department of Life, Health and Environmental Sciences, University of L’Aquila, L’Aquila, Italy; ^3^Department of Mental Health, Local Health Agency of the National Health System, L’Aquila, Italy

**Keywords:** online social network, mental health, quality of life, post-disaster

## Abstract

**Background:** An understudied area of interest in post-disaster public health is individuals’ use of social networks as a potential determinant of quality of life (QOL) and mental health outcomes. A population-based cross-sectional study was carried out to examine whether continual use of online social networking (Facebook) in an adult population following a massive earthquake was correlated with prevalence of depression and post-traumatic stress disorders (PTSD) and QOL outcomes.

**Methods:** Participants were a sample of 890 adults aged 25–54 who had been exposed to the L’Aquila earthquake of 2009. Definition of “user” required a daily connection to the Facebook online social network for more than 1 h per day from at least 2 years. Depression and PTSD were assessed using the Screening Questionnaire for Disaster Mental Health. QOL outcomes were measured using the World Health Organisation Quality of Life BREF (WHOQOL-BREF) instrument. Logistic regression was carried out to calculate the prevalence odds ratios (POR) for social network use and other covariates.

**Results:** Two hundred and twenty one of 423 (52.2%) men, and 195 of 383 (50.9%) women, had been using Facebook as social network for at least 2 years prior to our assessment. Social network use correlated with both depression and PTSD, after adjusting for gender. A halved risk of depression was found in users vs. non-users (POR 0.50 ± 0.16). Similarly, a halved risk of PTSD in users vs. non-users (POR 0.47 ± 0.14) was found. Both men and women using online social networks had significantly higher QOL scores in the psychological and social domains of the WHOQOL-BREF.

**Conclusion:** Social network use among adults 25–54 years old has a positive impact on mental health and QOL outcomes in the years following a disaster. The use of social networks may be an important tool for coping with the mental health outcomes of disruptive natural disasters, helping to maintain, if not improve, QOL in terms of social relationships and psychological distress.

## Introduction

Natural disasters have both short and long-term health effects, both on quality of life (QOL) outcomes (1, 2) and the prevalence of mental health disorders like depression and post-traumatic stress disorders (PTSD) and functional disabilities (3, 4). QOL is an outcome measure that has evolved to include aspects of life that affect patients’ perceived physical or mental health, and it is a fundamental measure used to understand a population’s health status in post-disaster environments, where QOL is threatened by radical changes in daily lifestyle, habits, and resources. Post-disaster QOL and mental health assessments are important for understanding how the population reacts to the initial effects of a natural disaster, and to understanding its aftermath (5). It is worth noting that prevalence estimates of mental health outcomes after natural disaster are strictly dependent from the type of disaster and the extent of the interested area. Studies of PTSD after natural disasters include persons from a broader area affected by the disaster, possibly including persons who were less directly exposed. Therefore, prevalence estimates after natural disaster report a PTSD prevalence ranging from approximately 5–60% in the first 2 years from the disaster (4).

An understudied area of interest in post-disaster public health is individuals’ use of social networks as a potential determinant of QOL and mental health outcomes.

Social networks are online venues for information sharing and social communication, where users create public profiles, interact with friends, and connect with other users (6). Social networks have expanded quickly over the past decade, such as Facebook, which currently has 1.3 billion users, of whom 50% log into Facebook daily (7). Furthermore, the total time spent on Facebook by the average user has increased by 500% per year (8), which indicates the exponential appeal of online social networks. The first internet-based communities emerging during the 1990s were based on the shared interests of their members, whereas web 2.0 social networks are more focused on user-generated content and expression.

By permitting the free and public communication of personal and intimate statuses, with potential feedback from contacts and intuitive measures of approval (“likes”), social networks fulfill a basic human need for social connection. In this way, social networking has been hypothesized to widely affecting health at an individual level, by modifying individual’s approach to health (9), improving decision making in very specific clinical conditions (10), or enhancing the effects of pharmacotherapy on psychological impairments (11). However, some research also suggests that rather than enhancing well-being, use of social networks may undermine well-being (12), cause networking addiction (8), or increase the prevalence of depression in adolescents (13). Thus, evidence regarding the impact of social network use remains uncertain and controversial.

However, following disasters, online social networks may offer potential psychological benefits for vulnerable populations, gained through their collective participation as stakeholders in the response. Disaster victims report a psychological need to contribute, and by doing so, they are better able to cope with their own situation. As well, affected populations may gain resilience by replacing their helplessness with dignity and control, along with personal and collective responsibility, perceived connectivity to others, and increased self-esteem (14).

In 2009, an earthquake of magnitude 6.3 on the Richter scale devastated the city of L’Aquila, the capital of the Abruzzo Region of Italy. Three hundred and nine people were confirmed dead, and more than 2000 were seriously injured, making it one of the most severe natural disasters in Italian history. It was the first disruptive earthquake in recent European history to have an urban area of ancient historical importance as its epicenter. The earthquake spread across 5000 km^2^, and left at least 55,000 of the city’s 70,000 residents without housing, forcing them to live in tents or seek accommodations at the region’s coast hotels. The earthquake also destroyed the historical center of the town, including a significant number of businesses and public services, such as hospitals, outpatient and rehabilitation centers, and grade schools. The Italian government reported official estimates of direct economic losses and reconstruction costs at €10,000,000,000. The entire community experienced material, social, and psychological damages, and any sense of security and normalcy was further undermined by frequent aftershocks. Both mental health and QOL outcomes of the L’Aquila population have been described in terms of structural, process, and outcome perspectives in the aftermath of the earthquake (2–15). Three years after the earthquake, only 50% of the population had returned to their homes, while more than 15,000 persons were still living in provisional housing facilities on the town’s outskirts, with no correlation between previous residence or neighborhood and new housing assignation. Moreover, the town’s population density diminished dramatically, as the old city had a major axis of no more than 2 km, while the post-disaster population has been scattered over a roughly circular territory with a diameter of approximately 30 km. As a result, the social structure of the town has been destroyed, and most previous personal, familial, professional, and friendship relationships were dismantled. In this situation, social networks were quickly assumed into the daily life of the L’Aquila population, including age strata that do not typically use web 2.0 applications, as they presented an easy way to maintain or replace previous social relationships. Social networks may also have played role in resilience strategies, by increasing the “social capital” (i.e., features of social life such as networks, norms, and trust) that enabled participants to act together to more effectively pursue shared objectives.

### Objective

The aim of this study was to evaluate continual and intensive social network use as a potential determinant of mental health and QOL outcomes in the adult population of L’Aquila (age 25–54 years) after the disruptive earthquake of 2009.

## Materials and Methods

### Design

A population-based cross-sectional study was carried out to determine if continual use of online social networks for at least 2 years was correlated with depression, PTSD, and adverse QOL outcomes in an adult population previously exposed to a massive earthquake.

### Participants

From January to December 2013, a representative random sample of 890 adult inhabitants of L’Aquila (Italy) between the age of 25 and 54 were recruited, comprising 3% of the 2012 general population of the municipality (target population *N* = 29,660) (16). Participants had been directly exposed to the earthquake in 2009 and were invited to participate in a screening program for mental health and QOL, as part of the post-earthquake population programs promoted by the local Department of Mental Health. Eight hundred and six of 890 (90.5%) participated in the screening, including 383 of 450 women (85%) and 423 of 440 men (96%). Each participant provided written informed consent; the study was approved by the advisory board of the Department of Mental Health of L’Aquila Health Agency and was conducted according to the Helsinki Declaration.

### Outcome and explanatory variables

Mental health outcomes were measured by administering the Screening Questionnaire for Disaster Mental Health (SQD) (17, 18), an efficient and easy-to-use instrument for epidemiologic and public health screening in the aftermath of natural disasters. The SQD was originally developed after the Kobe 1995 earthquake in Japan, comprised interview-format simple screening questions, and can be implemented in brief interviews. The SQD was developed as an instrument for use in situations where time is restricted and respondent burden must be minimized, such as in post-disaster epidemiological surveys. Since it is an individual-centered instrument, designed for use as a multi-dimensional profile, the SQD enables a wide range of conditions to be compared. Questions are mostly related to assessing the meaning of different aspects of the respondents’ lives, and how satisfactory or problematic their experiences are. In diverse populations, and especially if senior citizens or those with minimal education are included, it is crucial that the questionnaires’ items are easy to understand. Since these people are typically reluctant or feel uncomfortable in completing self-reporting scales, the SQD interview format is much more preferable. Moreover, it consists of only 12 yes/no questions and has the advantage of being easily incorporated into local post-disaster services, where non-experts in mental health can administer it after appropriate training. SQD detects scores of both depression (SQD-D score, ranging from 0 to 6) and PTSD (SQD-P score, ranging from 0 to 6). The possible outcomes for SQD-D are “less likely to be depressed” (scores from 0 to 4) and “more likely to be depressed” (scores of 5 and 6). In our study, depression was defined as SQD-D ≥ 5. The possible outcomes for SQD-P are “slightly affected with little current possibility of PTSD” (scores between 0 and 3), “moderately affected” (scores of 4 and 5), and “severely affected with possible PTSD” (score 6). We chose to dichotomize the PTSD outcomes (0–3 vs. 4–6), enabling a simpler interpretation where the moderately and severely affected (SQD-P score ≥ 4) were all defined as cases of PTSD.

Quality of life outcomes were measured using the Italian version of the World Health Organisation Quality of Life BREF (WHOQOL-BREF) standardized instrument (19, 20), developed for use in epidemiological surveys encompassing health-related and contextual issues, along with general health and overall QOL. The WHOQOL-BREF provides an assessment of QOL in four domains: physical health (seven items), psychological (six items), social relationships (three items), and environmental (eight items). Two further items examine the subjective scoring of overall QOL and health. Each item contributes to the calculation of the overall domain score, ranging from 0 to 100, which enables comparisons across domains.

The major explanatory exposure variable was defined as daily social network use that had begun at least 2 years before the questionnaire was administered. Our definition of “user” required a daily connection to online social networks, namely Facebook, for more than 1 h per day: otherwise, participants were defined as non-users. Employment (employed vs. unemployed), housing (property or tenancy residence vs. provisional housing), and cohabitation (living together vs. single) were selected as possible explanatory covariates.

### Data analysis

Our analysis considered gender and three 10-year age strata (25–34, 35–44, and 45–54 years) as potential covariates. Descriptive statistics for the SQD outcomes were reported as prevalence estimates of depression and PTSD among participants across all covariate strata. Logistic regression was carried out to calculate prevalence odds ratios (POR) for online social network use and other covariates as potential determinants for depression and PTSD occurrence (21). For each covariate, a likelihood ratio analysis was set for model fitting, assessing the weight of the covariates in terms of odds ratios, while holding all other covariates constant. Wald tests, adjusted for multiple testing, were used to assess the statistical significance of covariates, which was set at 5%. Both explanatory and response variables, where necessary, were transformed into dummy variables. Such a transformation enabled us to set the contribution of each category to SQD-D and SQD-P responses. The role of social network exposure in modifying the overall QOL profile, structured according to the four WHOQOL-BREF dimension vectors, was tested using a preliminary Hotelling *T*^2^ test. Afterward, *t*-test comparisons detected the statistically significant dimensions of WHOQOL-BREF. All analyses were performed using the statistical software STATA version 13 (StataCorp LP, College Station, TX, USA).

## Results

Table 1 includes the descriptive characteristics of participants. Two hundred and twenty one of 423 (52.2%) men, and 195 of 383 (50.9%) women, had been using Facebook as social network for at least 2 years prior to our assessment: it is worth noting that 15% of all Facebook users were also Twitter users. In agreement with the typical profile of regular social media users, our users were predominantly younger men (54–67%) and women (50–60%), compared to only 33.6% men and 39.1% women in the 45–54-year-old stratum. Regarding employment status, this study included 476 employed (59%) and 330 unemployed (41%) subjects. It is notable that more than two-thirds of men were employed across all age strata, whereas the proportion of employed women was 47–50% and 38% in 25–44 and 45–54 years strata, respectively. Regarding housing status, 366 (45.4%) subjects were living in a permanent or tenancy residence, and 440 (54.6%) were living in provisional welfare housing, with no significant differences between the age strata. Regarding cohabitation status, 468 (60.1%) subjects were living together and 338 (39.9%) were single, with significant differences in both men and women between the age strata (approximately 70% of 45–54-year-old men and women were married, compared with approximately 45% of 25–44-year-old subjects).

**Table 1:** **Descriptive characteristics of participants**.

	Explanatory variables							
	Online social networks use		Employment		Housing		Cohabitation status	
	User	Non-user	Employed	Unemployed	Residence (property or tenancy)	Provisional welfare housing	Living together	Single
**Males**
25–34 years	87 (66.9)	43 (33.1)	91 (70.0)	39 (30.0)	56 (43.1)	74 (56.9)	58 (44.6)	72 (55.4)
35–44 years	93 (54.4)	78 (45.6)	127 (74.3)	44 (25.7)	80 (46.8)	91 (53.2)	101 (59.0)	70 (41.0)
45–54 years	41 (33.6)	81 (66.4)	82 (67.2)	40 (32.8)	51 (41.8)	71 (58.2)	85 (69.7)	37 (30.3)
**Females**
25–34 years	75 (60.0)	50 (40.0)	63 (50.4)	62 (49.6)	57 (45.6)	68 (54.4)	58 (46.4)	67 (53.6)
35–44 years	84 (50.6)	82 (49.4)	78 (47.0)	88 (53.0)	76 (45.8)	90 (54.2)	99 (59.6)	67 (40.4)
45–54 years	36 (39.1)	56 (60.9)	35 (38.0)	57 (62.0)	46 (50.0)	46 (50.0)	67 (72.8)	25 (27.2)

*Cell values: No. (%)*.

Table 2 includes the mental health outcomes across gender and age strata. Overall, 138 of 383 (36.0%) women were classified as more likely to be depressed, compared to 87 of 423 (20.5%) men, and 131 of 383 (34.2%) of women were classified as moderately or severely affected by PTSD, compared to 118 of 423 (27.9%) men. Prevalence of depression was higher in 45–54-year-old men (28.7%) compared to the first two age strata (16.4–18.5%), whereas in women, both the second and third age strata exhibited a higher prevalence (39.2–43.5%) compared to the first stratum (26.4%).

**Table 2:** **SQD mental health outcomes (depression, PTSD) by gender and age strata**.

		SQD-D Depression		SQD-P PTSD	
		More likely to be depressed	Less likely to be depressed	Moderately or severely affected	Slightly affected
Males	25–34 years	24 (18.5)	106 (81.5)	22 (16.9)	108 (83.1)
	35–44 years	28 (16.4)	143 (83.6)	43 (25.1)	128 (74.9)
	45–54 years	35 (28.7)	87 (71.3)	53 (43.4)	69 (22.6)
Females	25–34 years	33 (26.4)	92 (73.6)	29 (23.2)	96 (76.8)
	35–44 years	65 (39.2)	101 (60.8)	66 (39.8)	100 (60.2)
	45–54 years	40 (43.5)	52 (56.5)	36 (39.1)	56 (60.9)

*Cell values: No. (%)*.

Taking into account the association between social network use and mental health outcomes (Table 3), overall prevalence of depression was significantly lower in users (45 of 416, 10.8%) vs. non-users (173 of 390, 44.3%), after adjusting for gender (Cochran-Mantel-Haenszel chi-square test *p* < 0.0001). Likewise, overall prevalence of PTSD in social network users (87/416, 20.9%) was statistically lower vs. non-users (162 of 390, 41.5%), after adjusting for gender (Cochran-Mantel-Haenszel chi-square test *p* < 0.0001).

**Table 3:** **SQD mental health outcomes (depression, PTSD) by online social network use and gender**.

	More likely to be depressed	Less likely to be depressed	
**Depression^a^**
Males
Users	20	201	221
Non-users	67	135	202
	87	336	423
Females
Users	25	170	195
Non-users	113	75	188
	138	245	383

	**Moderately or severely affected**	**Slightly or not affected**	

**Ptsd^b^**
Males
Users	44	177	221
Non-users	74	128	202
	118	305	423
Females
Users	43	152	195
Non-users	88	100	188
	131	252	383

*^a^Cochran-Mantel-Haenszel chi-square test = 123.1; *p* < 0.0001*.

*^b^Cochran-Mantel-Haenszel chi-square test = 38.9; *p* < 0.0001*.

Using our logistic regression analysis, adjusted for potential covariates, model fitting was significant for both outcomes (depression: 168.67, *p* < 0.05; PTSD: 50.62, *p* < 0.05). Our analysis (Table 4) found a statistically significant difference in the POR for social network users compared non-users, using both SQD-D and SQD-P binary response variables (SQD-D POR = 0.50 ± 0.16; SQD-P POR = 0.47 ± 0.14). The POR for depression was significantly lower among social network users compared to non-users, and the POR for PTSD was lower among social network users compared to non-users. A logistic analysis, examining social network use, showed an 11% reduction in the POR for depression among men using social networks, compared to non-users $\left({\text{POR}}_{\text{User}}^{\text{SQD}-\text{D}}(\text{Gender})=0.43\pm 0.16\;\text{vs}.\;{\text{POR}}_{\text{Non}-\text{user}}^{\text{SQD}-\text{D}}(\text{Gender})=0.54\pm 0.12\right)$. This result was not detected for PTSD.

**Table 4:** **Prevalence odds ratio estimations for mental health outcomes by explanatory variables**.

		SQD-D POR ± SE	*p* > |*z*|	SQD-P POR ± SE	*p* > |*z*|
Online social network use	Non-user	1.00^a^	0.029	1.00^a^	0.010
	User	0.50 ± 0.16		0.47 ± 0.14	
Gender	F	1.00^a^	0.001	1.00^a^	n.s.
	M	0.50 ± 0.09		0.91 ± 0.22	
Age	25–44 years	1.00^a^	0.001	1.00^a^	0.045
	45–54 years	1.22 ± 0.07		1.12 ± 0.09	
Working condition	Unemployed	1.00^a^	0.001	1.00^a^	0.022
	Employed	0.37 ± 0.11		0.53 ± 0.25	
Habitation	Provisional	1.00^a^	0.04	1.00^a^	n.s.
	Resident	0.53 ± 0.17		1.01 ± 0.15	

*^a^Reference category*.

Regarding covariates, male gender and employment status were potential protective determinants for both depression (POR_male_ = 0.50 ± 0.09, POR_employed_ = 0.37 ± 0.11) and PTSD (POR_male_ = 0.70 ± 0.12, POR_employed_ = 0.53 ± 0.15). Older age (45–54) significantly increased the risk of depression (POR = 1.22 ± 0.07), although it was also a significant risk protector for PTSD (POR = 0.70 ± 0.12). Cohabitation status was not statistically significant for any of our measured outcomes.

Table 5 includes the results of our Hotelling’s *T*^2^ test, which were consistent with the effect of social network use on QOL (*T*^2^ = 38.41, *p* < 0.05). Pairwise comparisons yielded statistically significant differences at 5% for type I error in the WHOQOL-BREF psychological and social dimensions, with higher scores in female users vs. female non-users (psychological dimension: 74.07 ± 4.24 vs. 67.88 ± 4.48, *p* < 0.05; social dimension: 73.12 ± 4.37 vs. 70.10 ± 4.49, *p* < 0.05), and in female users vs. male users (psychological dimension: 74.07 ± 4.24 vs. 67.73 ± 6.06, *p* < 0.05; social dimension: 73.12 ± 4.37 vs. 70.84 ± 3.94, *p* < 0.05).

**Table 5:** **Dimensional WHOQOL-BREF mean scores by gender and online social network use**.

	Online social network use	*n*	WHOQOL-BREF dimensions (Mean ± SD)			
			Physical	Psychological	Social	Environmental
Women	User	195	67.69 ± 5.78	74.07 ± 4.24^a^^,^^b^	73.12 ± 4.37^c^^,^^d^	70.91 ± 6.44
	non-user	188	67.68 ± 5.75	67.88 ± 4.48^a^	70.10 ± 4.49^c^	71.39 ± 5.78
Men	User	221	68.82 ± 5.76	67.73 ± 6.06^b^	70.84 ± 3.94^d^	70.24 ± 4.94
	non-user	202	67.88 ± 5.43	68.58 ± 6.67	71.38 ± 4.23	69.28 ± 5.21

*^a,b,c,d^Pairwise comparisons with *p* < 0.05; all other comparisons were not significant*.

## Discussion

To our best knowledge, this is the first study with an epidemiological design that addresses the potential role of social network use as a determinant of mental health and QOL outcomes in post-disaster environments. Social networks, such as Facebook and Twitter, are rapidly changing the way people interact and communicate all over the world, and evidence is mounting regarding their impact on public health, as well as specific health domains such as general medicine and mental health. In post-disaster environments, online social network use assumes a very special role, especially when routine personal relationships are completely upset, as was the case in L’Aquila. The literature has emphasized the information sharing role of social networks in post-disaster environments, where involved people are transformed from content consumers into content producers (14). Following this perspective, our study examined the effect of social network use on mental health and QOL outcomes at a population scale, using adults aged 25–54 years.

We deliberately chose to exclude younger subjects (i.e., people < 25 years) from eligible participants, in order to avoid any bias derived from their well-known overexposure to social media. Moreover, children and adolescents require special attention after a major disruptive event, and indicators of their well-being or health status are strongly dependent on how they are evaluated. As well, social network use is essentially ubiquitous in this age group, which would preclude comparison between users and non-users. We also chose to exclude the elderly from our analysis, as it is well known that in Italy and other Latin countries, aged subjects are less likely to use web 2.0 applications and social media, with the exception of technologically skilled subjects (who are in the minority). Therefore, data obtained from this age group would be essentially irrelevant for our purposes.

Our study population (25–54 years) can be considered the most active group in responding to a disaster, as they must care for dependents (both young and elderly), while maintaining work activities and making housing arrangements. Although subjects of this age group are in majority social network users, a considerable proportion remain non-users, allowing us to accurately compare the two groups in terms of explanatory and outcome measures.

Our observed mental health outcomes, as determined by the SQD screening instrument, were consistent with previous reports of the prevalence of depression and PTSD in populations affected by disasters (22). Depression occurs in approximately 30% of 45–54-year-old men, compared to 15–20% of 25–44-year-old men. The same is true for PTSD, which occurs in more than 40% of 45–54-year-old men, compared to approximately 15–25% of 25–44-year-old men. In women older than 35 years, the prevalence of high risk of depression and PTSD was approximately 40–45% and 40%, respectively. In terms of covariate-adjusted POR, a significantly higher risk of depression (+22%) and PTSD (+12%) was found in the elderly, compared to younger age groups.

Social network use was strongly associated with both depression and PTSD, after adjusting for gender. Expressed in terms of POR, we observed a halved risk of depression in users vs. non-users (POR 0.50 ± 0.16), which is strongly suggestive of a role for social network use in preventing depression. Our prevalence data for depression, in relation to social network use and gender are striking: in men, depression occurred in 20 of 221 (9%) social network users, compared to 67 of 202 (33.1%) non-users. In women, depression occurred in 25 of 195 (12.8%) social network users, compared to 113 of 188 (60.1%) non-users. Similar results were observed for PTSD, with a halved risk in users vs. non-users (POR 0.47 ± 0.14). Similarly, prevalence data for PTSD deserves special notice: in men, PTSD occurred in 44 of 221 (20%) users, compared to 74 of 202 (36.6%) non-users, whereas in women, PTSD occurred in 43 of 195 (22%) users, compared to 88 of 188 (46.8%) non-users. In terms of adjusted multivariable analysis, employment and permanent housing were potential determinants of positive mental health outcomes, which are consistent with the literature (23). Regarding QOL outcomes, our results suggest that both male and female social network users have significantly higher QOL scores in the psychological and social domains, compared to non-users. The finding of decreased mental health negative outcomes and increased QOL in the psychological and social domains in social network users suggests, consistently with the literature (24) that PTSD and depression are strongly related with QOL after an earthquake.

The limitations of this study include the cross-sectional design and lack of data regarding the previous mental health and QOL of the participants. Cross-sectional or prevalence studies are often employed for public health planning, and are also suitable for examining risk factors for diseases of slow onset and long duration, such as mental disorders. As well, since it is difficult to diagnose the specific onset of mental disorders, case-control or cohort studies are difficult to design. A major advantage of cross-sectional studies is that they examine a sample of the general population and are not biased toward subjects seeking medical care, and thus their generalization may be considered a strength. Regarding the current study’s lack of longitudinal data regarding QOL and mental health outcomes, the statistically significant association (expressed in terms of POR) between social network use and mental health outcomes must be considered in light of the cross-sectional nature of the design. However, we favor an interpretation in terms of a causal effect, as our data are consistent with prospective studies in other settings.

Facebook activity may be an indicator of a person’s psychological health. Some studies have suggested that social media use may even improve mental health and well-being: people who share fewer pictures on the site communicate less frequently, have a longer profile and fewer Facebook friends, and are more likely to experience social anhedonia, i.e., the inability to encounter happiness from activities that are normally enjoyable, such as talking to friends (25). Another study suggests that using social media may even spread happiness: happy status updates encourage other users to post happy status updates themselves. Emotional expressions spread online and also positive expressions spread more than negative (26).

In conclusion, while risk modeling is limited by the cross-sectional design, our results suggest that social network use may be a determinant of positive mental health and QOL outcomes following a natural disaster, at least in the population of adults 25–54 years old. Although we recommend further longitudinal research, the results of this study are sufficiently powered to conclude that the use of social networks appears to be a strong tool for facing mental health sequelae of disruptive disasters. In these situations, social network use maintains, if not improves, QOL in terms of social relationships and psychological distress, and we encourage policies at the local level aiming at spreading the use of social networks after a disruptive disaster.

## Author Contributions

Francesco Masedu and Marco Valenti conceived, designed, and co-ordinated the epidemiological study, performed the statistical analyses, and drafted the manuscript. Monica Mazza contributed to data analysis, interpretation of psychometric dimensions of the instruments and contributed substantially to the drafting of the manuscript. Sergio Tiberti and Vittorio Sconci participated in the design, implementation, and co-ordination of the study, and contributed substantially to data interpretation and the drafting of the manuscript. Chiara Di Giovanni and Anna Calvarese contributed to instrument administration, to the evaluation of WHOQOL-BREF and SQD scores, and to the drafting of the manuscript.

## Conflict of Interest Statement

The authors declare that the research was conducted in the absence of any commercial or financial relationships that could be construed as a potential conflict of interest.
